# Key successes and challenges in providing mental health care in an urban male remand prison: a qualitative study

**DOI:** 10.1007/s00127-016-1170-2

**Published:** 2016-02-04

**Authors:** Chiara Samele, Andrew Forrester, Norman Urquía, Gareth Hopkin

**Affiliations:** Informed Thinking, 10 Grove Road, London, SW19 1BL UK; Institute of Psychiatry, Psychology and Neuroscience, King’s College London, 16 De Crespigny Park, London, SE5 8AF UK; South London and Maudsley NHS Foundation Trust, 108 Landor Road, London, SW9 9NT UK; Health Service and Population Research Department, Institute of Psychiatry, Psychology and Neuroscience, King’s College London, 16 De Crespigny Park, London, SE5 8AF UK

**Keywords:** Prison mental healthcare, Integration, Multi-agency, In-reach, Prisoner

## Abstract

**Purpose:**

This study aimed to describe the workings of an urban male remand prison mental health service exploring the key challenges and successes, levels of integration and collaboration with other services.

**Method:**

A purposive sampling was used to recruit key prison and healthcare professionals for in-depth interviews. A thematic analysis was used to analyse transcripts based on an initial coding frame of several predefined themes. Other key themes were also identified.

**Results:**

Twenty-eight interviews were conducted. Prisoners referred to the service had complex, sometimes acute mental illness requiring specialist assessment and treatment. Key successes of the in-reach service included the introduction of an open referral system, locating a mental health nurse at reception to screen all new prisoners and a zoning system to prioritise urgent or non-urgent cases. Achieving an integrated system of healthcare was challenging because of the numerous internal and external services operating across the prison, a highly transient population, limited time and space to deliver services and difficulties with providing inpatient care (e.g., establishing the criteria for admission and managing patient flow). Collaborative working between prison and healthcare staff was required to enable best care for prisoners.

**Conclusions:**

The prison mental health in-reach service worked well in assessing and prioritising those who required specialist mental health care. Although the challenges of working within the prison context limited what the in-reach team could achieve. Further work was needed to improve the unit environment and how best to target and deliver inpatient care within the prison.

## Introduction

The introduction of prison in-reach mental health services and the transfer of responsibility for healthcare from prisons to the National Health Service marked an important step to improving the mental healthcare of prisoners in England and Wales [1, 2].

A key ambition in the modernisation of prison healthcare was to provide equivalent healthcare to that available in the community [1]; although this has proved inadequate given that prisoners present with greater and more complex mental healthcare needs when they are compared with community samples [3, 4]. Estimates of the prevalence of mental health problems, such as psychosis, personality disorder, dual diagnosis (mental illness and substance misuse) and depression, in prisoners have consistently shown these to be far higher than those found in the general population [5, 6]. For example, the prevalence of personality disorder was 66 % among prisoners across England and 5.3 % in the general population [7, 8]. Similarly high rates of treatable mental health problems have also been found in prisoners across the world [9]. Globally, prisoner population rates have increased considerably over the past 15 years from 136 to 144 per 100,000 population over the past 15 years [10]; and from 41,800 prisoners in June 1993 to 85,786 by October 2014 in England and Wales [11, 12]. This increase is likely to have further intensified the demand for prison mental healthcare.

Several studies in the UK have found wide variation in models of prison in-reach services and their operational characteristics which have been described as limited and idiosyncratic [13–16]. One national survey of in-reach teams found a 20 % increase in their size between 2004 and 2007, but despite this expansion demands for the service continued to increase [17].

The scale of the problem has been further highlighted in a more recent study in which 23 % of the 3492 prisoners screened using research tools had a severe mental health problem; where 25 % with SMI, had been assessed by in-reach teams and only 13 % were then accepted onto their caseloads for further work [18]. In-reach teams are unlikely to be able to manage the high magnitude of mental health needs in prisons alone, which indicates a need to improve existing screening mechanisms [19, 20].

Primary healthcare care (PHC) in prisons provide medical consultations, referral to secondary healthcare and other services that are supposed to be equivalent to those provided by general practices in the community. The integration of in-reach services with PHC was another important move to improving mental healthcare in prison. However, integration has proceeded at different paces in different prisons, and has, to some extent, been dependent upon how these services are commissioned locally and the arrangements for providing these health services in prison.

The majority of referrals to in-reach teams have been found to come from PHC, however triage by PHC was considered by in-reach leads in one survey to be poor because of a lack of expertise and resources and worked less well in local prisons [17]. However, in-reach team leaders also reported liaison with health and social services outside the prison (e.g., mental health services in the community, primary and social care services) was also found to be problematic, where certain groups of prisoners were felt to be considered less sympathetically by these external agencies [17].

Alongside prison in-reach services some prisons also operate health-care wings or inpatient units to provide front-line mental illness triaging and care for complex individuals who display challenging behaviour. These prison health-care wings are comparatively less well documented, yet manage very high levels of disturbance among prisoners who are acutely unwell. According to one service evaluation over a 20 week period, 88 prisoners were admitted to the health-care wing of a busy local male remand prison [21]. Over a quarter of those admitted needed hospital transfer and 11 required emergency compulsory treatment in prison.

Despite the available literature on the prevalence of mental illness in the prison population and national service based research, there remains a limited understanding of the experience of local teams and how and what might work, particularly around integrated and collaborative working.

This study aims to describe the workings of a prison in-reach mental health service exploring the key challenges, successes and levels of integration and collaboration with other services.

## Method

### Setting

The service evaluation was carried out in a category B Local remand prison for men situated in a densely populated area of South London. During the study period the ethnically diverse prison’s population varied between 750 and 800 prisoners and had a high population turnover.

### Procedure

A purposive sampling was used to recruit key prison and healthcare professionals for in-depth interviews. Thirty-five key staff were identified for interview. Staff were recruited with respect to whether they currently worked within the prison’s health and mental health service, or did so in the previous year, had been involved in setting up the mental health service, managed the service, or liaised with it from outside the prison, such as forensic mental health staff who facilitated transfer to hospital. This created the opportunity to explore the diverse way in which the service was delivered to prisoners with mental health problems.

Interviews lasted between 30 min and 1 h. Semi-structured interview guides were developed to explore the different components of the prison mental health service—the in-reach service and inpatient unit.

Interviews were carried out between March and June 2012. Where permitted interviews were audio recorded and transcribed verbatim. Handwritten notes were taken for two interviews where an audio recorder could not be used.

### Participants

A total of 28 professionals—from prison to health/mental health care staff—were recruited and interviewed, 20 of whom were male and the remainder female. Table 1 lists the number of those interviewed by job title and gender.

**Table 1:** Number, job title and gender of prison and health care staff interviewed

Mental health professionals and managers (*n* = 27)	
Psychiatrists, including visiting consultant	5 (4 male, 1 female)
Psychiatric nurses, including team lead	7 (all male)
Psychologist and team lead	2 (1 male, 1 female)
Administrator	1 (male)
Occupational therapist	1 (female)
Clinical service manager	1 (female)
NHS trust service lead and project manager	2 (1 male, 1 female)
General healthcare	
Private healthcare manager	1 (male)
Primary care	
Primary care mental health team nurses	2 (1 male, 1 female)
Lead general practitioner	1 (male)
Substance misuse service	
Substance misuse team leader	1 (male)
Prison staff	
Deputy governor	1 (female)
Wing governor (inpatient unit)	1 (male)
Senior prison officer (inpatient unit)	1 (male)

### Service evaluation approval

The study was granted service evaluation approval from the South London and Maudsley NHS Foundation Trust Clinical Effectiveness Committee (Ref no. 38).

### Analysis

A thematic analysis was used to analyse transcripts [22]. The analysis was guided by the main aims of the project and used to develop an initial coding frame of several predefined themes—referral, triaging, in-reach activity, collaboration, prisoner profiles, challenges and successes. Two raters (CS and NU) coded the data according to predefined themes and identified patterns in the data for further themes or interpretations. The coding frame was checked for appropriateness through subsequent iterations of analysis. An interview summary sheet was used to contain the data from the themes. The analysis was conducted using the summary sheets.

NVivo Version 9 (2010) was use to index and retrieve data for the codes interpreted from the data.

## Results

The mental health in-reach service was first established in 2002 and provided by an NHS team working full-time within the prison. The service worked as a Community Mental Health Team (CMHT) to provide treatment and support for a mental health problem by a specialist mental health worker. The team comprised three full-time mental health nurses, part-time administrative support, part-time clinical psychology input and sessional support from consultant and staff grade psychiatrists.

In 2008, a private healthcare contractor was commissioned to provide general healthcare to the prison; who in turn subcontracted mental health services from the local Mental Health NHS Trust.

### Screening, referrals and assessment

An open referral system was adopted in 2008, allowing all referrals from any source from both within and external to the prison, including self-referral, cell-mates and families for assessment, follow-up and treatment by the in-reach service. The in-reach team’s ethos was that no referral should ever be considered inappropriate. This allowed in-reach services to be more accessible than that found in the community, where access to NHS specialist mental health services is largely gate-kept through GP (general practitioner) referral. A mental health nurse was located at the prison reception desk and would triage all new prisoners, which was important for ensuring appropriate referrals to the in-reach service. A standardised health screening tool [23] was used to detect mental health problems. Anyone with previous contact with mental health services was usually noted. Referral from the reception screening staff was the most common source of referral [24]. Referrals also came from the primary mental healthcare (PMHC) team (who provide treatment and support for prisoners with mild to moderate mental health conditions) and prison officers. The open referral system was viewed as an important featured of how the in-reach service worked:

I think the open referral system is…I suppose it’s almost like the feather in the cap really of how in-reach works. (Locum Consultant Psychiatrist)

The referral pathways into the team… are more open than the pathways into community mental health services, and that’s because there’s an open referral policy, so we will accept referrals from prison officers, we’ll accept referrals from pharmacists, you know, because sometimes they will refer, you know, I mean…not that I think it’s ever happened, but if one of the cleaners for example, you know, wanted to refer, they could (Locum Consultant Psychiatrist).

Up to 12 referrals were received each week by the prison in-reach team; nine of whom were accepted into the caseload. There were around six prisoners on the in-reach caseload who were transferred to other prisons and three discharges per week to mental health services in the community.

There was a 48-h turnaround for an initial assessment. Assessments were conducted by the in-reach team on a daily basis and this sometimes impacted on the time for seeing people on existing caseloads. Even two assessments proved difficult to do alongside usual liaison work because these usually required considerable time in seeking out information about the person’s history and any previous treatment, which had to be fully documented.

Understanding who should be treated by PHC and in-reach staff was not always clear:

…With there being so many services, in effect, discipline staff or prisoners themselves coming by self-referral or prison staff, and it’s do they refer to in-reach or primary care? Sometimes it’s difficult for a non-healthcare person (to decide); it’s difficult enough for a healthcare person! (Healthcare Manager).

This was not a common issue but did lead to some inevitable tensions between relevant staff. This grey area also included any treatment initiated by PHC.

### Further assessment, risk management and treatment

The in-reach team kept a caseload of around sixty people and used a red, amber and green zoning system to assess risk and manage their caseload of people with serious and enduring mental illness. Those at highest risk were rated red and followed up more frequently. The most complex, difficult and acute cases were transferred to the inpatient unit while awaiting admission to outside hospital. This zoning system was regarded as another important success in the way in-reach services worked. It offered a very practical way of planning and managing caseloads. The zoning system also aided the management of referrals.

Treatment for prisoners with existing mental illness was not always just a simple continuation of what the prisoner may have been receiving in the community or from PHC within the prison. At times existing diagnosis and medication had to be reviewed by the in-reach team:

Most of the people we get are already diagnosed, so when they come in we just contact their CMHT outside for the history of mental health. We have to continue their treatment and maybe they’ll (the CMHT) will send us a package of information or we access the EPJS (electronic case record system). We’ve had so many scenarios where some of them (prisoners) are on treatment but I don’t see why they should be on that because they’ve been discharged. You get so many of them (prisoners) who say they have schizophrenia. Maybe they are on remission and their treatment has to change, maybe a lower dose, of what they said they are on (In-reach psychiatric nurse).

The prison regime meant the in-reach team had a very limited window of opportunity to carry out its assessments and treatment, usually between 1 and 2 h a day. If a prisoner was deemed high risk and required three officers present when their cell was unlocked this too became challenging if not enough officers were available. The limited space in the prison, particularly the lack of interview rooms, further exacerbated this issue.

In-reach staff used a Care Programme Approach (CPA)—a system used to organise care from specialist mental health services [25]. The high turnover of remand prisoners, however, made using CPA in all cases challenging:

The problem is these are remand prisoners in and out of court. Those that were sentenced are easy to arrange a CPA for because we know exactly when they would get released and we’d arrange a care package ready for his release. But remand prisoners getting released from court, pulled everybody short really and no CPA was arranged, so we had to advise the CMHT, that due to that reason, can not be done, so we’d either send a letter to the CMHT or speak to them (In-reach psychiatric nurse).

### Integrated working

Multi-agency integration particularly between the prison and in-reach staff was especially important for preventing deaths in custody. After a spate of deaths senior management staff at the prison prioritised the integration of healthcare/mental health staff much more into the wider prison; and even attempting to involve them in the running of the prison:

*…* whether it be getting them to (prison) staff briefings, making sure that you do small things like if there’s a performance recognition, that you’re always thinking about the staff on the healthcare side as well; all of those kinds of things in terms of being able to include them (Deputy Governor).

For 4 years the prison had no deaths in custody and this success was attributed to improvements in prison healthcare, better communication and integration of services generally.

I think that’s a significant measure of how far (the prison) has come, and a more reflective approach about previous deaths in custody. We tried to learn about what hadn’t worked so well before and I think that not having a death for that amount of time was a really remarkable achievement, it took a huge amount of effort (Wing Governor, Healthcare).

The integration between health and prison services therefore was seen as fundamental to preventing deaths in custody. Working collaboratively was deemed important, although this was created in part through the introduction of the Prison’s Service’s ACCT (Assessment, Care in Custody and Teamwork procedures) strategy on suicide risk management. The ACCT aims to bring together staff from all disciplines to prevent and reduce suicide and self-harm in prison [26].

The formal introduction of the ACCT to assist in preventing suicide and self-harm brought together prison and health care staff and helped facilitate more integrated and collaborative working within the prison. Including healthcare staff in prison briefings also promoted a better understanding of what was required to improve mental health care. In practice, the ACCT involved an intensive amount of recording, monitoring and multi-agency reviews which on a daily basis was difficult to carry out, especially if more than 20 prisoners were being managed on it.

However, PMHC and the in-reach service operated separately as they were delivered by different providers:

Working with in-reach…I feel that we’re quite separate teams in a sense that my understanding is that we deal with primary care with what you would deem first level mental health problems, and they (in-reach) would be dealing with more severe and acute mental illness. So they refer to us and we refer to them but we do liaise on a regular basis (Primary Care Mental Health Nurse).

Team meetings between these mental health care services were also separate, with only occasional invitations to discuss cases. The PMHC saw a much more transient population sometimes with brief, one-off consultations. Some cases remained on the caseload for longer than was necessary but would be discharged and referred on to other through care services such as IAPT (Improving access to psychological therapy). The healthcare wing tended to refer to the PMHC as part of their discharge pathway and this was perceived as challenging particularly if the referral included someone with very complex issues, such as personality disorder.

Integrated working with external agencies coming into the prison was also challenging. Keeping track of the external services coming into the prison to deliver health related care was also difficult for those overseeing prison healthcare:

I don’t know the names of them now but there’s IAPT, there’s End to End for the substance misuse part; there’s several, there’s quite a few, too many for me to remember. But they’ll come in; they’ll deliver sessions or groups or be involved in that way… (Healthcare manager).

External services, however, provide an important resource for the prison and CMHT staff are often invited to CPA meetings to discuss cases. The process for bringing external staff into the prison has become much easier in recent years enabling better access to community services while a person is in prison and later on release.

### Inpatient care

The inpatient unit had 25 beds and was originally intended to be a temporary holding bay while prisoners awaited transfer to hospital [21]. Two of the bed spaces had constant supervision/24-h watch. The average number of admissions and discharges was around one per day. Those admitted were usually those with very challenging behaviour and many had intense and acute psychopathology. Sometimes any challenging behaviour was not always directly related to mental health problems and personality disorder was a common issue.

We’ve had groups of prisoners in here (the inpatient unit) who might have a personality disorder of one sort or another, they might have a type of mental illness, they might just be badly behaved and trying to convince everyone that they’re mentally ill, and sometimes it’s been sometime before the clinical staff could be sure that they did not fit the criteria, they did not require inpatient, and that it’s been clear that it’s been a behavioural issue; they’re just badly behaved (Healthcare unit, Wing Governor).

Considerable concerns were raised about the provision of acute inpatient care in the prison, particularly by mental health staff. Yet, the demand for this was high and managing patient flow and who was suitable for admission made this a challenging area for all staff involved. The prison appeared to need a safe place for some prisoners who were difficult to manage on ordinary wings. Some of these were deemed high risk and required a relatively speedy assessment to identify any mental health issues, which was not always possible.

The unit environment was considered inadequate by many staff. Space was limited and the unit was considered not fit for purpose:

The inpatient unit, I do not think works well at all. There have been several issues. One was it was not purpose built, it was temporary and we were going to have a new build, but…mental health inpatient units in prisons…no matter how many beds you’ve got, you’ll fill them (Healthcare manager).

The inpatient unit had a multi-purpose function to meet the needs of the prison managing those with challenging behaviour; provide acute inpatient care as part of the mental health in-reach service; and manage a small number of prisoners with physical health problems, for example broken limb or tuberculosis. Prison staff would consult senior clinical support before attempting to admit a prisoner to the unit. Although outside working hours this was not always possible which created some tension.

## Discussion

Several themes, either pre-defined or interpreted from the data highlighted some of the successes of the in-reach service based in a remand prison, including the open referral system, locating a mental health nurse at reception to screen all new prisoners, the zoning system to prioritise urgent or non-urgent cases and pursuing an integrated and collaborative service. The key challenges for a mental health in-reach service included attempts to achieve integrated healthcare because of the numerous internal and external services operating across the prison, the limited time and space to deliver services and the provision of inpatient care (e.g., the criteria for admission). An integrated model of offender healthcare, however across other health and social care areas, particularly outside the prison offers an important opportunity to manage prisoners with complex needs, but requires sufficient resources to ensure effective and sustained improvements to offenders’ mental health [27, 28].

A key success was the adoption of an open referral system and a psychiatric nurse conducting screening assessments at reception. This was confirmed in an evaluation of this reception screening service which found significantly more suitable referrals to the in-reach team and little evidence of ‘mission creep’ whereby specialist services absorb primary care level mental health problems [19].

This is an important stage by which to identify mental health issues in prisoners and initiate treatment early on; as is secondary screening for prisoners at ultra high risk of psychosis [29]. However, the recommendations following a screening assessment are usually governed by the mental health resources available in a prison [30].

A study of referrals to the same in-reach team over an 18-week period in 2008/2009 found that around a quarter accessed services in prison for the first time, while about a third were actively at risk of self-harm/suicide; although foreign national prisoners were under-referred, and there were very few self-referrals [24]. Despite its success the open referral system and a 48-h turnaround for nurse triage assessments appeared to impact on the time available for seeing those on the existing in-reach caseload and shows perhaps where mental health resources were limited. This is not unusual; often in-reach services are not always sufficiently resourced to meet the high demand for them [17]. However, applying a zoning system in which to prioritise need helped manage this to some extent in terms of the level of care available. Another study of the same prison found that some prisoners however were missed at first screening, where a further 3 % of a cohort of assessed prisoners was later picked up by the in-reach service who had developed a first episode of psychosis [29].

Establishing clear criteria for whom in-reach teams should assess and treat is important so that clinical need can be addressed appropriately. Decision-making regarding the level of care is not always consistently associated with the clinical characteristics of prisoners’ with mental illness according to one study as those who received PHC were more likely to have a diagnosis of major depressive disorder, and those with a diagnosis of psychosis were more likely to receive secondary mental health services [31]. Given the complexity of need found among people in prison it is not always easy to assess and treat, so ensuring that triage is improved or works appropriately therefore is essential for meeting clinical need [31].

Triaging via PHC has not been considered viable due to the lack of resources and expertise; even though these services are the largest source of referral to in-reach teams [17]. And indeed, an open referral system is, in some ways, intended to bypass PHC triage, avoiding delays which are inevitably introduced by gate-keeping.

Placing all those admitted to the inpatient unit on an ACCT notably increased the workload of both prison and healthcare staff and so impacted on the time needed for other essential activities. However, this system has the capacity to enhance multi-agency integration and improve the safety of prisoners through preventing suicides [32].

Concerns about the standard of inpatient care in prisons have been debated to some degree in the limited literature available, particularly in relation to hospital transfers and the most appropriate care pathways for this process [21, 33, 34]. A key concern was the inpatient unit itself which was not considered suitable or appropriate to meet the needs of acutely mentally ill prisoners. Over a decade earlier an inspection programme of inpatient facilities in 13 prisons found the quality of service fell well below standards in the NHS [35]. Prison inpatient facilities have improved considerably since then. However, there continues to be a high degree of variability across establishments with some prison inpatient units being old and others new and purpose built.

### Limitations of the study

The study was conducted just before the prison was rerolled from a Category B remand prison to a Category C (both categories are defined as closed or locked prisons but vary slightly in terms of prisoners’ likelihood of escape). This meant many changes were about to take place and staff were uncertain about their future. The changes included a reduction in numbers of the mental health in-reach team and closure of the inpatient unit. This may have influenced some of the responses given during interviews and the exact descriptions and workings of the prison in-reach service. However, staff were briefed about describe the working of the prison mental health services prior to the changes in the prison. This was taken into consideration both during interviews and as part of the analyses. Some interviews were not recorded because of restrictions on the use of a digital recorder in prison. Instead notes were taken and it was not possible to capture all the information expressed in some depth interviews with key healthcare professionals. Some professionals may not have felt comfortable expressing some of the more difficult aspects of delivering mental healthcare within the prison context and so descriptions and working relationships may be more positive than was actually the case. Nevertheless many participants did detail the main challenges encountered.

## Conclusions

The in-reach service assessed delivered mental health services to a busy local remand prison for men. Prisoners referred to the service had complex, sometimes acute mental illness requiring specialist assessment and treatment. The key areas of success included the open referral system which allowed referrals from a range of sources, including self-referral. However, at times this led to an increased workload for the in-reach team in trying to assess new referrals and meet the clinical needs of prisoners on existing caseloads. Time and space limited what in-reach staff were able to do with prisoners requiring support. This was further hampered by working with an often transient population who could disappear at little if any notice.

Prison and healthcare staff working in a collaborative and integrated way was essential not least for meeting the monitoring requirements for prisoners on an ACCT which aimed to prevent/reduce suicide and self-harm.

The prison’s inpatient unit posed some key challenges, where the criteria for admission were sometimes blurred for this relatively costly resource which included constant watch/24 h supervision.

These findings provide important lessons about delivering and improving mental healthcare in prison, particularly inpatient care which is often wholly inadequate for treating very high levels of acuity. Further work was needed to improve the inpatient environment and how best to target and deliver inpatient care within the prison.
